# Enhancing the Urea-N Use Efficiency in Maize (*Zea mays*) Cultivation on Acid Soils Amended with Zeolite and TSP

**DOI:** 10.1100/tsw.2008.68

**Published:** 2008-04-20

**Authors:** Osumanu H. Ahmed, Aminuddin Hussin, Husni M. H. Ahmad, Anuar A. Rahim, Nik Muhamad Abd. Majid

**Affiliations:** ^1^ Department of Crop Science, Faculty of Agriculture and Food Sciences, Bintulu Campus, Universiti Putra Malaysia, 97008 Bintulu, Sarawak, Malaysia; ^2^ Department of Land Management, Faculty of Agriculture, Universiti Putra Malaysia, 43400 UPM, Serdang, Selangor, Malaysia; ^3^ Department of Forest Science, Faculty of Agriculture and Food Sciences, Bintulu Campus, Universiti Putra Malaysia, 97008 Bintulu, Sarawak, Malaysia

**Keywords:** ammonia volatilization, zeolite, triple superphosphate, urea, urea-N uptake efficiency, soil exchangeable ammonium, nitrate, acid soils, maize cultivation

## Abstract

Ammonia loss significantly reduces the urea-N use efficiency in crop production. Efforts to reduce this problem are mostly laboratory oriented. This paper reports the effects of urea amended with triple superphosphate (TSP) and zeolite (Clinoptilolite) on soil pH, nitrate, exchangeable ammonium, dry matter production, N uptake, fresh cob production, and urea-N uptake efficiency in maize (*Zea mays*) cultivation on an acid soil in actual field conditions. Urea-amended TSP and zeolite treatments and urea only (urea without additives) did not have long-term effect on soil pH and accumulation of soil exchangeable ammonium and nitrate. Treatments with higher amounts of TSP and zeolite significantly increased the dry matter (stem and leaf) production of Swan (test crop). All the treatments had no significant effect on urea-N concentration in the leaf and stem of the test crop. In terms of urea-N uptake in the leaf and stem tissues of Swan, only the treatment with the highest amount of TSP and zeolite significantly increased urea-N uptake in the leaf of the test crop. Irrespective of treatment, fresh cob production was statistically not different. However, all the treatments with additives improved urea-N uptake efficiency compared to urea without additives or amendment. This suggests that urea amended with TSP and zeolite has a potential of reducing ammonia loss from surface-applied urea.

## INTRODUCTION

Ammonia loss significantly reduces urea-N use efficiency in crop production[1,2]. Efforts to reduce ammonia loss are mainly laboratory oriented. Reduction in urea-N use efficiency in agriculture, particularly when urea is surface-applied to soils, has been generally associated with ammonia volatilization and it is thought to be the major pathway for urea-N loss from the surface-applied urea[1,2].

Ammonia volatilization in acid soils is generally thought to occur because of high pH and exchangeable ammonium concentrations in the microsite immediately around the fertilizer[3]. In acid soils, triple superphosphate (TSP) has been used to reduce ammonia loss[3] because it makes the microsite immediately around the fertilizer acidic[3]. The high cation exchange capacity (CEC) and great affinity for ammonium ions of humic acids and zeolite have enabled the use of these materials to reduce ammonia volatilization[3,4,5,6,7]. The small internal tunnels of clinoptilolite zeolite, as an example, have been found to physically protect ammonium ions from high nitrification by microorganisms[8]. This process does not only reduce ammonia loss, but it also helps in releasing ammonium ions slowly into the soil[9,10].

It must be stressed that most of the studies were carried out under laboratory conditions; as such, the results may not reflect actual field conditions. A field study was carried out to evaluate the effects of urea amended with TSP and zeolite on soil pH, soil nitrate, soil exchangeable ammonium, dry matter production (leaf and stem), N uptake, fresh cob production, and urea-N uptake efficiency in maize cultivation in actual field conditions on an acid soil. Positive effects on these variables should lead to reduction in ammonia loss and should, in turn, improve the urea-N use efficiency from surface-applied urea.

## MATERIALS AND METHODS

The study was conducted on a sandy clay loam Typic Kanduidult (Bungor Series) of the University Putra Malaysia Agricultural Farm at Puchong. The experimental area has annual precipitation of about 2,200 mm. This area also has a mean monthly maximum and minimum temperature of 32 and 24°C, respectively, and a relative humidity between 70 and 90%. The study was carried out between November 2006 and March 2007. The experimental field of the study was a cultivated field that had received little fertilization. The experimental design was a randomized complete block design with three replications (blocks). The plot size within each block was 1.5 m (length) × 1.5 m (breadth). The distance between plots was 1 m and that between blocks was 1.5 m. The planting distance was 0.75 m between rows and 0.25 m within plants. The pH, CEC, nitrate, and exchangeable ammonium of the experimental plots prior to the application of treatments (i.e., before planting or starting the experiment) were statistically not different. There were 18 plants in each plot.

The treatments evaluated were (1) normal N, P, K application (74.34 g urea, 27.36 g TSP, 24.12 g KCl) (T1), (2) urea-TSP mixture (74.34 g urea + 27.36 g TSP) + 24.12 g KCl (T2), (3) 74.34 g urea + 27.36 g TSP + 9.0 g zeolite (T3), (4) 74.34 g urea + 27.36 g TSP + 13.5 g zeolite (T4), and (5) no fertilization (T5). Note, the same amount of 24.12 g KCl was used in T3 and T4 plots. The amounts of urea, TSP, and KCl used were based on the standard recommendation for the test crop (Swan). The zeolite rates were adopted, based on several laboratory trials; they gave better mixtures[5]. It must be noted that the rates used in this study were a scale up from our previous laboratory trials. Treatments 3 and 4 were prepared by first weighing the three materials (for each treatment) separately into plastic vials. The materials were then transferred into a set of plastic vials, tightly closed, and shaken on a reciprocal shaker at 150 rpm for 30 min to ensure that they were uniformly mixed.

Ten days after planting (DAP), T1, T2, T3, and T4 were applied to the test plots. At 28 DAP, T1, T2, T3, and T4 were again applied. The plants were monitored and harvested (excluding guard rows) at 90 DAP. The weight of the harvested cobs was taken *in situ* using a weighing balance. Harvested stems and leaves were oven dried at 60°C until constant weight was attained. Afterwards, their weights were also taken by using a weighing balance. Before planting and a day before harvesting, composite soil samples (bulk of three samples) were taken from 0–20 cm depth in each of the experimental plots. The soil samples were air dried and ground to pass a 2-mm sieve.

Standard procedures were used to determine the selected chemical properties of zeolite, soil, TSP, and urea. The pH of the urea, zeolite, soil, and TSP were determined in a 1:2.5 soil:distilled water suspension and/or 0.01 *N* $CaCl_2$ using a glass electrode[11]. The CEC of the zeolite was determined by the CsCl method[12]. Soil CEC was determined by leaching with 1 *N* ammonium acetate buffer adjusted to pH 7.0 followed by steam distillation[13]. Soil samples at harvest were analyzed for pH using the method previously outlined. Exchangeable ammonium and available nitrate at harvest were extracted from the soil samples by the method of Keeney and Nelson[14], and the amount determined using a LACHAT Autoanalyzer (LACHAT Instruments, Milwaukee, WI). Total N of the plant tissues (stem and leaf) was determined by the Micro-Kjeldhal method[13]. Analysis of variance was conducted to test for treatment effect, while means of treatments were compared using Tukey's test[15]. Urea-N use efficiency was calculated according to the following formula[16]:

$$\% \text{ fertilizer nutrient recovery} = (\text{TNF}) - (\text{TNU})/\text{R} \times 100$$

where TNF = total nutrient uptake from fertilized plots, TNU = total nutrient uptake from unfertilized plots, and R = rate of fertilizer nutrient applied.

## RESULTS AND DISCUSSION

The soil pH (water; 1:2.5 soil:distilled water suspension), pH (0.01 *N* $CaCl_2$), and CEC before planting were 4.50, 4.00, and 6.40 cmol $kg^{-1}$, respectively. These results were consistent with those reported by Paramananthan[17]. The pH (water), pH (0.01 *N* $CaCl_2$), and CEC of zeolite were 6.10, 5.30, and 105 cmol $kg^{-1}$, respectively, and they were also comparable with those reported by Prama[18], who also provided other properties of this zeolite. Irrespective of treatment, the soil pH (water), pH (0.01 *N* $CaCl_2$), exchangeable ammonium, and available nitrate at harvest were not significantly different at $p = 0.05$ (Table 1). This observation is consistent with the finding of a study that evaluated these treatments on Bungor Series cultivated with a maize variety called Putra J-56[19], suggesting that T2, T3, and T4 have no long-term effect in controlling the release of these nutrients under field conditions.

**TABLE 1**
**Soil pH, Soil Available Nitrate, and Soil Exchangeable Ammonium at 90 DAP**

| Treatment | $pH_{(1\ N\ KCl)}$ | $pH_{(water)}$ | $NO_3$ (ppm) | $NH_4$ (ppm) |
|---|---|---|---|---|
| T1 | 3.86 | 5.09 | 7.97 | 609 |
| T2 | 3.79 | 5.18 | 7.77 | 563 |
| T3 | 3.83 | 5.09 | 7.77 | 653 |
| T4 | 3.88 | 5.30 | 7.93 | 544 |
| T5 | 3.93 | 5.50 | 5.80 | 470 |

In one of our laboratory studies on the effect of urea amended with TSP and zeolite, we found significant accumulation of ammonium at only 0–3 cm after 15 days of incubation[7]. We found no significant accumulation of ammonium at 3–7.5 cm, while for nitrate, regardless of soil depth, there was no significant accumulation irrespective of the treatment[7]. In the case of pH, our laboratory study results also indicated no significant effect regardless of the treatment and soil depth after 15 days of incubation[7]. The pH of the TSP was 2.50 and was considered low, but higher than that reported by Sample and Soper[20]. This difference may be partly attributed to the acceptable amounts of K, Ca, and Mg in the TSP[5]. The pH of the urea was 8.00 and it was also considered high.

Compared to T5, T3 and T4 (treatments with higher amounts of TSP and zeolite) significantly increased the dry matter production (stem and leaf) of the test crop. In a related field study, urea with the highest amount of additives significantly increased the stem and leaf dry weight of Putra J-56 maize variety[19] (Table 2). All the treatments had no significant effect on the urea-N concentration in the leaf and stem of the test crop, while in terms of urea-N uptake in these parts, only T4 significantly increased urea-N uptake in the leaf of the crop (Tables 3 and 4).

**TABLE 2**
**Dry Weight of Stem and Leaf of Swan at 90 DAP**

| Treatment | Stem | Leaf |
|---|---|---|
| | (g plant$^{-1}$) | |
| T1 | 156.60$^{bc}$ | 58.27$^{abc}$ |
| T2 | 127.45$^{bc}$ | 49.89$^{bc}$ |
| T3 | 164.74$^{ab}$ | 93.27$^{a}$ |
| T4 | 203.51$^{a}$ | 85.89$^{ab}$ |
| T5 | 123.01$^{c}$ | 36.32$^{c}$ |

Note: Different alphabets within column indicate significant difference between means using Tukey's test at $p$ = 0.05.

**TABLE 3**
**Urea-N Concentrations in Stem and Leaf of Swan at 90 DAP**

| Treatment | Stem | Leaf |
|---|---|---|
| | (%) | |
| T1 | 0.83 | 2.40 |
| T2 | 1.37 | 2.70 |
| T3 | 1.13 | 2.10 |
| T4 | 1.35 | 2.50 |
| T5 | 1.13 | 1.90 |

**TABLE 4**
**Urea-N Uptake in Leaf and Stem of Swan at 90 DAP**

| Treatment | Leaf | Stem |
|---|---|---|
| | (g plant$^{-1}$) | |
| T1 | 1.40$^{ab}$ | 1.30 |
| T2 | 1.30$^{ab}$ | 2.38 |
| T3 | 1.90$^{ab}$ | 2.03 |
| T4 | 2.40$^{a}$ | 2.34 |
| T5 | 0.80$^{b}$ | 1.29 |

Irrespective of treatment, fresh cob production was statistically not different (Table 5), but in terms of efficiency, T2, T3, and T4 improved urea-N uptake efficiency (Table 6). It is interesting to note that, under T1 (urea without additives), the overall urea-N use efficiency was only 9%, while those under T2, T3, and T4 (urea with additives) were approximately 20, 17, and 31%, respectively. Based on the climatic information provided, perhaps the additives of these treatments may have caused a temporary reduction in ammonia loss at the initial stages of the application of the treatments compared to T1.

**TABLE 5**
**Fresh Cob Yields of Swan at 90 DAP**

| Treatment | kg Ten Plants$^{-1}$ |
|---|---|
| T1 | 3.38 |
| T2 | 3.37 |
| T3 | 3.48 |
| T4 | 3.42 |
| T5 | 3.00 |

**TABLE 6**
**Urea-N Uptake Efficiency at 90 DAP**

| Treatment | Leaf | Stem | Total |
|---|---|---|---|
| | | (%) | |
| T1 | 7.90 | 0.18 | 8.87 |
| T2 | 6.70 | 13.50 | 20.20 |
| T3 | 7.90 | 9.00 | 16.90 |
| T4 | 17.90 | 13.00 | 30.90 |
| T5 | na | na | na |

na, not applicable.

## CONCLUSIONS

In terms of urea-N uptake in the leaf and stem tissues, only T4 had significant effect on urea-N uptake in the leaf of the test crop. Irrespective of treatment, fresh cob production was statistically not different. However, all the treatments with additives improved urea-N efficiency compared to urea without additives or amendments. This suggests that urea amended with TSP and zeolite has the potential of reducing ammonia loss from surface-applied urea. Hence, this could contribute to reduction of environmental pollution particularly in relation to urea use in agriculture.

## ACKNOWLEDGMENT

The authors are grateful to PETRONAS for financial and technical collaboration.
